# Semi-field evaluation of efficacy and residual activity of a microencapsulated pyriproxyfen formulation on *Anopheles arabiensis* emergence inhibition

**DOI:** 10.5281/zenodo.18649256

**Published:** 2026-02-15

**Authors:** Anitha Mutashobya, Augustino Thabiti Mmbaga, Simon Twaha Mnzava, Hulda Swai, Halfan Ngowo, Dickson Wilson Lwetoijera

**Affiliations:** 1 Environmental Health and Ecological Sciences Department, Ifakara Health Institute, P.O. Box 53, Ifakara, Tanzania.; 2 School of Life Sciences and Bioengineering, The Nelson Mandela African Institution of Sciences and Technology, P.O. Box 447, Arusha, Tanzania.

## Abstract

**Background:**

The integration of larviciding as a supplementary tool for malaria vector control requires effective larvicide formulations that are specifically suited to the target vector species, environmentally friendly, and cost-effective to reflect the needs of resource-limited settings in sub-Saharan Africa. This study evaluated the sublethal effect and its residual activity of two microencapsulated pyriproxyfen formulations containing 33% and 50% active ingredient (AI) against *Anopheles arabiensis* larvae exposed to sublethal doses.

**Materials and Methods:**

Sublethal effects on fitness parameters, including fecundity and wing length, were evaluated at concentrations that resulted in 20%, 50%, and 70% emergence inhibition, obtained from a dose-response curve. In three replicates across all three concentrations, fecundity was assessed in 25 adult females, while wing length was measured in 33 adult males and 33 adult females. Residual activity was assessed by exposing 200 3^rd^-instar larvae of *An. arabiensis* to each formulation at 0.06 mg/L 33% AI and 0.09 mg/L 50% AI in 5 replicates of artificial habitats. Once all mosquitoes had either emerged or died, a new batch of larvae was introduced every 2 weeks for 6 months.

**Results:**

Females emerged from concentrations inhibiting 70% of larval emergence exhibited significantly reduced fecundity; 88% for 33% AI [RR = 0.12, 95% CI: 0.11, 0.13, p < 0.001] and 85% for 50% AI [RR = 0.14, 95% CI: 0.07, 0.32, p < 0.001]. Adults emerged from sublethal concentrations had reduced body size, decreasing as concentration increased. Residual activity on emergence inhibition was 14.4% [13.2%, 15.6%], and 23.1% [18.7%, 27.5%], relative to 23% and 14% for 33% and 50% AI, respectively in the 6^th^ month. Control mortality was consistently below 10% during the entire evaluation period.

**Conclusion:**

These findings demonstrate the efficaciousness and long-lasting potential of microencapsulated pyriproxyfen formulations to target *An. arabiensis*, and highlight its consideration for application in larviciding programmes**.**

## Introduction

Globally, insecticide-treated bednets (ITNs) and indoor residual spraying (IRS) remain key interventions for malaria vector control [1]. These interventions have contributed to a substantial reduction in malaria-related cases and deaths, with ITNs alone contributing to an approximate 68% reduction in malaria cases in sub-Saharan Africa between 2000 and 2015 [2]. However, the rapid rise and widespread resistance of mosquitoes to pyrethroids, the core insecticides used in ITNs and IRS, alongside mosquito behavioural changes, such as preference to bite during early mornings and late evenings when people are often outdoors, threaten the effectiveness and future of these interventions [3]. These challenges have sustained the arms race for alternative pyrethroid chemistries, new vector control insecticides beyond pyrethroids, as well as other interventions such as spatial repellents and larval source management [4,5].

Larval Source Management (LSM) is the management of mosquito aquatic habitats, to prevent the completion of the development of the immature stages [1,6]. LSM is implemented to minimise vector densities by targeting mosquitoes at their aquatic stages, thereby making it a potential tool in controlling behavioural and insecticide-resistant mosquitoes [1,6]. Conventional larviciding, the most practiced form of LSM, has demonstrated historical successes, and is currently recommended for application in places with few, fixed and findable habitats [6]. This is due to LSM inefficient coverage of mosquito breeding habitats and high operational cost arising from the need for regular applications of larvicides with short persistence such as *Bacillus thuringiensis israelensis* (Bti) [7]. This necessitates improved and/or different formulations of larvicides, with relatively longer residual efficacy, including pyriproxyfen.

Pyriproxyfen (PPF), an insect growth regulator, is a juvenile hormone (JH) mimic that interrupts the normal development of aquatic-stage mosquitoes by inhibiting larvae-pupal metamorphosis [8]. It has low toxicity to non-target organisms and poses no risk to humans when used at or below the recommended dose of 300 parts per billion (ppb) for drinking water [9]. A key advantage of PPF is that no functional resistance has been reported among malaria vectors [10]. Additionally, it has long persistence, lasting from 2 weeks to 6 months [11,12].

Pyriproxyfen can be conventionally applied or disseminated by mosquitoes themselves through a process called autodissemination [13,14]. This technique utilises adult mosquitoes as the medium for carrying PPF from contaminated stations that act as resting habitats to their potential breeding sites, including those that are difficult for humans to find or access [15].

Previous studies under semi-field settings have demonstrated the potential of autodissemination with PPF to control different species of malaria vectors, notably *An. arabiensis*, *An. gambiae s.s.*, and *An. funestus* [16,17]. However, the use of autodissemination in field settings with expansive breeding habitats where contaminated stations may compete with numerous natural resting sites require innovative formulations, such as microencapsulated PPF [18]. This technique has several advantages over non-encapsulated PPF formulations including increased concentration of PPF into the breeding sites, due to increased numbers of particles transported by mosquitoes, which results from the electrostatic effect that increases PPF adherence to the mosquito body, fewer mosquitoes required for disseminating effective amounts of PPF, improved PPF stability, thereby extending its residual activity, and lastly reduction in operational cost as lower amounts of PPF and fewer treatment rounds will be needed [18,19]. This study summarises the efficacy and residual activity of two microencapsulated PPF formulations (33% and 50% active ingredient) against *An. arabiensis* under semi-field settings in southern Tanzania. The effect of PPF on emergence inhibition (EI), fecundity and body size fitness parameters following *An. arabiensis* exposure to sublethal doses, and their 6-months residual activities are presented.

## Materials and Methods

The study was conducted in a semi-field system (SFS) at Kining’ina village (8.10800°S, 36.66585°E) in rural Southern Tanzania between March and June 2025. The design and setup of the SFS for semi-controlled entomological studies has been previously described [20].

### Description of the test product

The two microencapsulated PPF formulations, 33% AI and 50% AI, that were evaluated are individually encapsulated within a polymeric, nanoporous microsphere carrier with electrostatic properties. The 33% formulation is highly porous (80%+) and lightweight, with the active ingredient bound within the polymer matrix, allowing for a slow, sustained release until depletion. In contrast, the 50% formulation has lower porosity (30%+), with PPF residue present on the surface and within the porous structure, resulting in faster release. The procedure of preparing the test concentration followed the WHO standardised procedures as outlined in their guidelines [9]. The formulations were supplied by Banfieldbio. Inc Woodinville, USA.

### Mosquitoes and test procedures

The study utilised insectary-reared 3^rd^ instar larvae of *An. arabiensis* from the established Ifakara colony. Detailed procedures for colony rearing and maintenance are described elsewhere [20,21]. The mosquitoes used in this experiment were PPF sensitive. The bioassay aimed to determine sublethal doses that inhibit adult emergence by 20% (IE20), 50% (IE50), and 70% (IE70). A log dose–response analysis was conducted to generate the dose-response curve, from which the sublethal concentrations were derived (Table 1). The experimental bioassays were conducted under a 12-hr light/dark (12L:12D) photoperiod, using plastic basins measuring 8.5 cm in height × 15 cm in diameter, with 2.5 L of water each. Each test concentration and control were replicated four times, whereby respective doses of PPF were added in the treatment groups and excluded in the control group. A total of 300 3^rd^ instar *An. arabiensis* larvae were then introduced in each basin. Throughout the bioassay, larvae were fed Tetramin® fish food twice daily (morning and evening). Every morning, basins were inspected and pupae were removed, counted, recorded, and transferred into 100 mL plastic cups placed inside 30×30×30 cm netting cages for adult emergence monitoring. Emerged adults were recorded the following day and maintained on 10% glucose solution, while dead pupae and adults were documented. The experimental bioassays were repeated three times to account for potential confounding factors.

**Table 1:** PPF concentrations used for evaluating sublethal effect on fitness parameters.

PPF Active Ingredient (AI) %	% Emergence inhibition	Concentration (mg/L)
33%	EI_20_	2.6xl0^-7^
	EI_50_	3.5xl0^-5^
	EI_70_	6.9X10^-4^
50%	EI_20_	6.0xl0^-7^
	EI_50_	5.3xl0^-5^
	EI_70_	8.6X10^-4^

### Assessing PPF sublethal effects on mosquito fecundity and body size

The adult mosquitoes from sublethal exposure bioassays, placed in 30×30×30 cm cages, were maintained on a 10% glucose solution for five days to allow for mating. From each test concentration and control cages, 40 female mosquitoes were randomly selected and transferred to 15×15×15 cm cages, starved for 12 hours and thereafter blood fed via human arm exposure for 15 minutes per feed. Mosquitoes were fed on two consecutive days to ensure that they were fully engorged. 72 Hrs post feeding, using a mouth aspirator, 25 female mosquitoes were randomly selected from each treatment and control groups and individually transferred into separate paper cups lined with wet cotton wool and filter paper to stimulate oviposition. Cotton pads soaked in 10% glucose solution were placed over the cups to provide additional nutrition. After every two days, approximately 7 ml of water was sprinkled to each cup filter paper to maintain oviposition simulation and appropriate moisture. Cups were inspected daily for egg-laying activity, and dead mosquitoes were removed. Mosquitoes that failed to lay eggs within eight days were killed. The number of eggs laid by each female was counted using a stereo microscope. As a proxy for mosquito body size, wing size was measured on a subsample of 99 males and 99 female mosquito that were 4 days old post emergence. Mosquitoes were anesthetised at -10ºC. for 7 min after which a single wing either left or right side was removed for measurement. Distilled water was used to fix the wings onto the slide, the length from the apical notch to the auxiliary margins was measured using a micrometer ruler under a stereo-microscope [22].

### Assessment of the residual activity of PPF formulations

To simulate the actual aquatic habitat of *An. arabiensis* fifteen plastic basins with diameter 41 cm spaced 1 m apart, were buried to the rim inside the semi-field chamber. 1.5 Kg of soil dug from the SFS chamber was added to each basin, followed by 8 L of tap water. The habitats were left for 48 hours to allow soil particles to settle and habitats to acclimatise. On the 3^rd^ day, 200 insectary-reared 3^rd^-instar larvae were introduced, and concentrations of PPF (0.09 mg/L for 50% AI and 0.06 mg/L for 33% AI) were added in respective habitats groups after 3 hours of larval acclimatisation.

Treatment allocation followed a lottery system, with five habitats serving as controls, five treated with 33% AI, and five treated with 50% AI. Netting material was placed over the basins to prevent emerging adult mosquitoes from escaping. Daily monitoring recorded all emerged live and dead mosquitoes, and dead pupae. To establish residual activity, new batches of 200 3^rd^ instar larvae were introduced every 2 weeks, for a period of 6 months. Adhering to similar monitoring procedures, pupae were removed from the basin habitats using a plastic pipette, recorded, placed in plastic cups containing 50 ml of water from their respective basin, and observed for adult emergence. Water in the habitats was replenished as needed to maintain a constant water volume.

### Data analysis

Data were organised with Microsoft Excel and its analysis was performed using R statistical software [23]. Prior analysis of fecundity and wing size, visual assessment of data distribution using histograms and Q–Q plots were conducted. For wing size, model residuals rather than raw data were inspected to verify assumptions of normality and homoscedasticity. A Shapiro–Wilk test confirmed that residuals from the wing size model were approximately normally distributed, whereas fecundity data exhibited overdispersion. Means and 95% CI were estimated as William’s means to account for skewness in the number of eggs laid per mosquito. A generalised linear mixed model (GLMM) was fitted using an lme4 package in R to assess the impact of different PPF concentrations on the fecundity of *An. arabiensis*. PPF concentration was included as a fixed effect, while replicates were treated as random effects to account for pseudo-replication and unexplained variation among replicate sets. The number of eggs (*Yi*) was modeled using a negative binomial distribution with a log-link function:

Yij∼Negbin(pij,r)

Additional analysis was done to assess the impact of varying concentrations on the wing sizes of *An. arabiensis* when exposed to 33% or 50% active ingredients. This linear mixed model treated wing size as the continuous response variable and test concentrations as fixed factors while replicate was included as a random term. Since the interest was not on the difference between active ingredients, the analysis for each type was done separately. The wing sizes (*Ti*) were modeled using a normal distribution with an identity link function:

Tij∼Normal(uij,σ2)

Model residuals were examined graphically to confirm normality and homogeneity of variances across concentration levels. Pairwise comparisons of estimated marginal means between concentration levels were performed using the emmeans package with Tukey’s adjustment for multiple comparisons.

Graphical representations were generated using the ggplot2 package in R. Predicted means and relative risks were reported, along with corresponding confidence intervals. To assess the residual activity of the PPF formulations over time, all replicates were pooled, then the proportion of emergence inhibition was summarised by experimental round and active ingredient (33% and 50% AI), including a control. Mean proportion values along with 95% confidence were calculated for each round-treatment combination.

## Results

### Sublethal effects of microencapsulated PPF on fecundity

Concentrations were labelled A, B, C, representing concentrations inhibiting adult emergence by 20%, 50% and 70%, respectively. While 33% and 50% represented their respective active ingredient A33-2.6 ×10-7 mg/L, B33- 3.5 × 10-5 mg/L, C33- 6.9 × 10-4 mg/L, A50- 6.08 × 10-7 mg/L, B50- 5.3 ×10-5 mg/L, C50- 8.6 × 10-4 mg/L.

**Figure 1:**
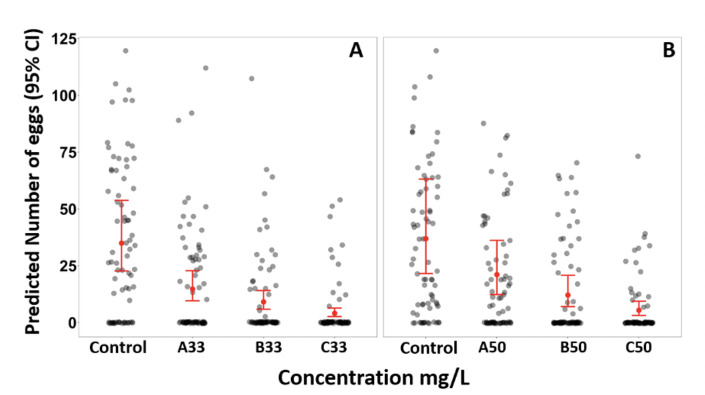
Predicted number of eggs laid by female *An. arabiensis* in the control group and those emerged from sublethal concentrations of microencapsulated PPF formulations. 33% AI (A) 50% AI (B).

PPF exposure adversely affected female mosquitoes in laying eggs as summarised in Figure 1 and Table 2. Mosquitoes that emerged in different concentration of PPF both 33% and 50% laid significantly fewer eggs compared to the control group. The results demonstrated a dose-dependent reduction in egg-laying behaviour across different concentrations of active ingredients (p < 0.001). The mean number of eggs (95% CI) laid in the control group was 34.9 (22.7, 53.8) eggs and 45.0 (26.3, 76.9) eggs for 33% and 50 % active ingredient respectively compared to 4.1 (2.6, 6.4) eggs and 6.7 (3.9, 11.5) eggs (p < 0.001 for all) of mosquitoes emerging from 70% emergence inhibition (IE_70_).

**Table 2:** Mean [95% CI] number of eggs laid by *An. arabiensis* in control and different percentage emergence inhibition of two active ingredients of microencapsulated formulations of PPF.

Active Ingredient	Concentration	Means [95% CI]	RR [95% CI]	P-value
33%	Control	34.9 [22.7, 53.8]	1	
	A33	14.8 [9.6, 22.8]	0.42 [0.40, 0.45]	< 0.001
	B33	9.1 [5.9,14.1]	0.26 [0.24, 0.28]	< 0.001
	C33	4.10 [2.6, 6.4]	0.12 [0.10, 0.13]	< 0.001
50%	Control	45.0 [26.3, 76.9]	1	
	A50	25.8 [15.1, 44.1]	0.57 [0.27, 1.21]	0.15
	B50	14.8 [8.7, 25.4]	0.33 [0.15, 0.70]	0.004
	C50	6.7 [3.9,11.5]	0.15 [0.07, 0.32]	< 0.001

The overall reduction of egg laying was 58% at A33, 74% at B33 and 88% at C33 for 33% AI and 43% at A50, 67% B55 and 85% at C50 for 50%.

### Sublethal effect of microencapsulated PPF on wing size

**Figure 2:**
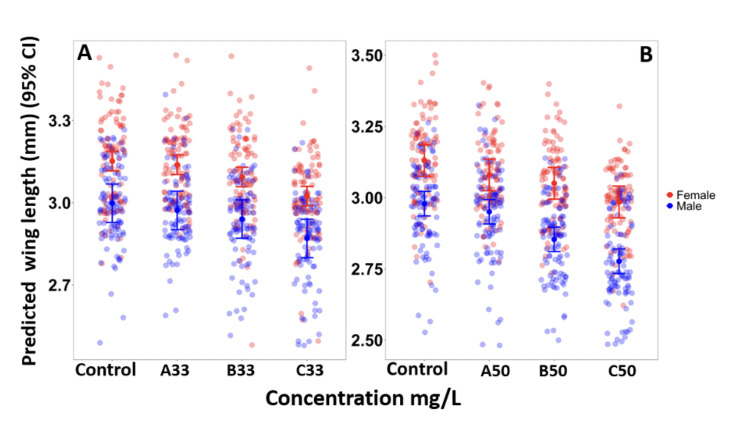
Predicted wing length male and female mosquitoes emerged in 33% AI (A) and emerged in 50% AI (B).

Evaluation of the sublethal effect of PPF on body size was assessed for both male and female mosquitoes. A total of 1,584 wings were measured and classified by sex for each PPF formulation. A distinct sexual dimorphism in wing size was observed, with females consistently having signifcantly larger wings than males across all treatments (Figure 2; Table 3).

**Table 3:** Pair-wise comparison of wing length between concentrations.

Active ingredient	Sex	Pair -wise comparison	t-ratio	p-value
33%	Males	A33 - B33	1.59	0.38
		A33 - C33	5.21	< 0.001
		B33 - C33	3.61	0.002
	Females	A33 - B33	2.10	0.15
		A33- C33	5.39	< 0.001
		B33 - C33	3.29	0.005
50%	Males	A50 - B50	4.60	< 0.001
		A50 - C50	8.27	< 0.001
		B50 - C50	3.64	0.002
	Females	A50 - B50	1.59	0.38
		A50 - C50	5.14	< 0.001
		B50 - C50	3.56	0.002

Mean (95% CI) wing length of male mosquitoes exposed to the 33% AI formulation had shorter wing length of 2.97 (2.90, 3.04) mm (p = 1.181) at A33 compared to the control group 2.99 (2.93, 3.03) mm. The mean wing length decreased with increase in concentration, where at B33 wing length was 2.94 (2.87, 3.01) mm (p = 0.003) and C33: 2.87 (2.79, 2.94) mm (p < 0.001) (Figure 2). A similar trend of smaller wing size was observed in exposed female mosquitoes, whereby A33 had 3.14 (3.10, 3.17) mm (p = 0.535), B33: 3.09 (3.06, 3.13) mm (p = 0.007), and C33: 3.02 (2.99, 3.06) mm (p < 0.001), compared to its respective control with mean wing length of 3.15 mm (Figure 2).

A comparable dose-response pattern was observed among mosquitoes exposed to the 50% AI formulation. Male mosquitoes in the control group had larger mean wing length of 2.98 (2.94, 3.02) mm, compared to exposed group with a mean wing length of 2.95 (2.91, 2.99) mm at A50 (p = 0.18), 2.85 mm (2.81, 2.90) (p < 0.001) at B50, 2.78 (2.73, 2.99) mm at C50 (p < 0.001). Female mosquitoes exhibited a similar trend, with control group wings measuring 3.13 (3.07, 3.19) mm compared to 3.08 mm (3.02, 3.14) at A50 (p = 0.007), 3.05 (2.99, 3.11) mm at B50 (p < 0.001), and 2.98 (2.93, 3.04) mm at C50 (p < 0.001) (Figure 2).

Pairwise comparisons of wing length across concentrations using Tukey’s HSD test revealed significant differences between concentrations within the same active ingredient (Table 3). For the 33% active ingredient, males exhibited a significant difference between A33 and C33 (t = 5.21 p < 0.001) as well as B33 and C33 (t = 3.61, p = 0.002), while A33 and B33 showed no significant difference (t = 1.59, p = 0.38). Similarly, for females, there was significant difference between A33 and C33 (t = 5.39, p < 0.001) and B33 and C33 (t = 3.29, p = 0.005), whereas A33 and B33 did not differ significantly (t = 2.10, p = 0.15).

For the 50% active ingredient, males exhibited significant differences in all comparisons: A50 versus B50 (t = 4.60, p < 0.001), A50 versus C50 (t = 8.27, p < 0.001), and B50 versus C50 (t = 3.64, p = 0.002). Females also showed significant difference between A50 and C50 (t = 5.14, p < 0.001) as well as B50 and C50 (t = 3.56, p = 0.002), while no signifcant difference was observed between A50 and B50 (t = 1.59, p = 0.38).

### Residual activity of PPF formulations on *An. arabiensis* emergence inhibition

**Figure 3:**
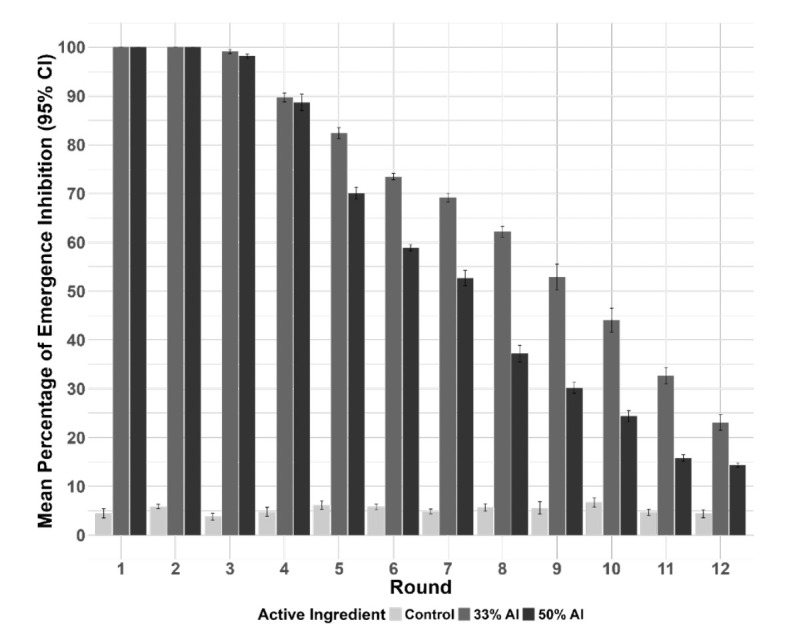
Mean percentage emergence inhibition (95% CI) of An. arabiensis exposed to 33% AI and 50% AI microencapsulated PPF formulation in the semi-field settings.

Residual activity of the two PPF formulations was done by pooling all replicates together within their respective formulation to calculate the mean percentage of adult emergence inhibition. The results, presented as mean (95% CI) percentage of adult emergence inhibition across all replicates, for both treated and untreated groups over 6 months is presented in Figure 3. Complete emergence inhibition (100%) was observed for both the 33% AI and 50% AI PPF formulations during the first 2 rounds (4 weeks post initial exposure) and by round 12 (approximately 6 months post initial exposure) the efficacy of the 50% AI in inhibiting emergence declined significantly to 14% (13.2%, 15.6%), and 23.1% (18.7%, 27.5%) at 33% AI, while percentage of emergence inhibition in the control group remained consistently under 10% throughout the experiment, which was two times below the recommended cut off defined by WHO [9].

## Discussion

This study documented reduced mosquito body size and fecundity for mosquitoes exposed to sublethal doses of PPF relative to unexposed mosquitoes. Microencapsulated PPF formulations had residual activities capable of resulting in more than 80% EI within the first two months to 50% within 3.5 months following initial treatment. The recorded differences between the fast-releasing 50% AI formulation and the slow-releasing 33% AI formulation can be explained by the formulation architecture that highlight their critical role in determining field efficacy and residual activity. The 50% AI formulation characterised by PPF residue coated on the surface and within the porous (30%+) microsphere structure shows higher bioavailability through a burst effect, yet it degrades more rapidly due to UV exposure and adhesion to organic matter. Conversely, the 33% AI formulation, in which PPF is embedded within a highly porous (80%+) slow‑degradable polymer matrix, provides gradual release and sustained inhibition over an extended period (as per the supplier’s manual). These findings on the sustained residual activity of the slow-release 33% AI formulation are consistent with previous research on Sumilarv® 0.5G, a similar slow-release PPF product, demonstrated its prolonged efficacy against *An. arabiensis* and *An. gambiae s.s.* [12]. Nayar *et al.* [24] reported that when applied to other mosquito species *Aedes aegypti*, *Ae. albopictus*, *Ae. taeniorhynchus*, *Culex nigripalpus* and *An. quadrimaculatus* Sumilarv® 0.5G achieved near-complete emergence inhibition for up to six weeks under standardised field conditions. Additionally, microencapsulation/controlled-release technologies have been successfully applied to other medically important arthropods for example, juvenile-hormone analogues and feed-through systems for sand flies [25], encapsulated repellents for tsetse control [26], and microencapsulated pyrethroid formulations used against fleas, ticks and other ectoparasites demonstrating that encapsulation can substantially extend residual activity and operational longevity across vector groups [27].

Beyond its lethal effects, PPF exposure induced negative physiological changes in the exposed mosquito population. In this study, sublethal concentrations of PPF led to measurable reductions in fecundity and body size, indicating developmental disruptions in affected mosquitoes. Fecundity was notably lower in mosquitoes that emerged from sublethal concentrations of PPF, suggesting potential interference with ovarian development and reproductive physiology. PPF acts as a juvenile hormone mimic, a key regulator of insect development, and its application during the 3^rd^ instar larval stage may have disrupted hormonal regulation, thereby interfering with oogenesis and vitellogenesis, ultimately reducing egg-laying capacity and overall egg production [28,29]. These findings align with recent studies demonstrating that PPF negatively affects reproductive traits in mosquitoes and other insects [12,30,31]. Additionally, the reduction in egg-laying observed in exposed females is likely linked to their relatively small body size. Previous studies have documented that larger mosquitoes tend to be more fecund, as they can ingest larger blood meals, supplying the nutrients necessary for egg production [28,32]. Furthermore, studies have shown that PPF can remain within adult mosquitoes for up to 5-6 days post-emergence. This internal persistence suggests that even sub-lethal doses acquired during the aquatic stages or through early adult contact continue to disrupt reproductive processes such as follicular development and egg viability well into the mosquito's adult life [33]. This prolonged hormonal disruption, combined with reduced body size, is likely a plausible explanation to the significant decline in fecundity observed in this study.

On the other hand, our study indicated that adult male and female mosquitoes emerging from sublethal concentrations had smaller body sizes compared to control groups and increased concentration exuberated the effect. Aldridge *et al.* [30] reported similar reductions in wing size in mosquitoes exposed to Nyguard (pyriproxyfen) at LC^50^ concentrations, while Moura *et al.* [31] found that sublethal PPF exposure led to smaller *Ae. aegypti* females compared to unexposed controls. This suggests that PPF disrupts mosquito development, likely through its juvenile hormone mimicry, which interferes with metamorphosis and adult emergence [28,34,35]. Other explainable factors for the smaller body size observed may include larval stress induced by PPF exposure. Such stress can disrupt normal development, leading to reduced growth [31]. Similar biological effects have been reported with *An. gambiae* exposed to sublethal concentrations of *Bacillus thuringiensis israelensis* (Bti), where larvicidal stress resulted in significantly smaller adults compared to unexposed counterparts [36].

PPF residual activity studies conducted in diverse conditions has displayed variable results ranging from 6 to 9 weeks inhibiting adult emergence of 80% that is recommended by WHO for ideal larvicides [12,37]. From this study the recommended efficacy of 80% was in the 8th and 10th week for 50% and 33% AI PPF formulations, respectively. It is difficult to compare the results among the studies considering the variables involved. The variation in documented residual activity across different studies might be due to differences in PPF formulation design and forms (either powders, granules, or microencapsulated), genetic background of the targeted mosquito species, and environmental factors [38,39]. The extended residual activity of up to 10 weeks exhibited by these formulation could be beneficial and practical for larviciding programmes [4,5]. These findings highlight the importance of selecting a formulation based on operational needs and mosquito ecology. Fast-releasing formulations are ideal for short-term interventions with strong early outcomes, whereas slow-releasing ones are better suited for sustained control in mosquito breeding sites. While field resistance to PPF in *Anopheles* mosquitoes has not yet been widely documented, its potential emergence poses a significant threat to future vector control efforts. The risk is underscored by the established history of PPF resistance in several agricultural pests and the recent detection of reduced susceptibility in *Ae. aegypti* populations [40]. As PPF-based interventions like dual active long-lasting insecticidal nets (LLINs) are deployed at scale, the frequent occurrence of sub-lethal residual doses resulting from natural insecticide degradation creates a selection environment conducive to the survival of resistant individuals. Given that metabolic enzymes like cytochrome P450s, which already confer resistance to other insecticide classes in *Anopheles* populations have been implicated in the degradation of PPF, proactive resistance monitoring and integrated management strategies are essential to preserve the efficacy of PPF in malaria-endemic regions [40].

It is important to note that our study only reported findings of sublethal exposure over a single generation not allowing for possible multi-generation effects. Moreover, residual activity was evaluated under semi-field conditions, which might not reflect the actual diverse habitats in the field.

## Conclusions

Microencapsulated PPF formulations demonstrated efficacy to reduce mosquito fecundity and body size, the critical traits for vectorial capacity. Extended residual activity for over 3 months for both microencapsulated formulations warrant consideration for practical and cost-effective larviciding programmes for malaria vector control, especially at this juncture of renewed momentum for larviciding. The findings highlight the need for further studies to evaluate the effectiveness of microencapsulated PPF formulations under varying field settings for different malaria vectors.
